# A comparative study of postural fixation techniques for radiotherapy in upper abdominal malignancies

**DOI:** 10.3389/fonc.2025.1625478

**Published:** 2025-09-12

**Authors:** Ji-Hua Han, Dong-Cheng He, Xiao-Ye Zhang, Yan Zhang, Jun Hong, Ting-Ting Shi, Zhi-Jian Zhu

**Affiliations:** Department of Radiation Oncology, The Affiliated Huaian No.1 People’s Hospital of Nanjing Medical University, Huai’an, China

**Keywords:** postural fixation, PTV margin, random error, setup error, systematic error

## Abstract

**Objective:**

In this study, we investigated the impact of two distinct postural fixation techniques on the incidence of setup errors in patients with upper abdominal malignancies.

**Methods:**

Seventy-seven patients with upper abdominal malignancies were divided into two groups: an observation group comprising 31 patients managed with a combination of thermoplastic body film and an air bag, and a control group consisting of 46 patients managed solely with thermoplastic body film. Prior to radiotherapy, a cone-beam computed tomography (CBCT) scan was performed, followed by registration of CBCT scans and positioning computed tomography (CT) images. Setup errors along the X (left/right), Y (superior/inferior), and Z (anterior/posterior) axes of the two groups were recorded and analyzed. A Wilcoxon rank-sum test was used for data analysis. Random errors (Σ), systematic errors (σ), and planning target volume (PTV) margins (MPTV) were evaluated.

**Results:**

The three-directional setup errors of the observation group [X: 1.9 (0.90, 2.73), Y: 2.5 (1.48, 3.7), Z: 1.4 (0.78, 2.3)] and the resultant displacement vector [T: 3.97 (3.08, 5.32)] exhibited lower magnitudes compared to those observed in the control group [X: 2.3 (1.1, 3.6), Y: 3.5 (2.1, 5.4), Z: 1.8 (0.9, 3.1); T: 5.51 (4.18, 7.25)]. These differences were statistically significant (p < 0.05). Notably, the Σ, σ, and MPTV in the observation group were consistently smaller than those observed in the control group.

**Conclusion:**

The combined use of thermoplastic body film and an air bag in postural fixation significantly reduces setup errors during radiotherapy for patients with upper abdominal tumors. This combined approach enhances the precision of postural alignment, thereby improving positional repeatability and reducing both random and systematic errors. Furthermore, this method is associated with decreased planning target volume margins, providing better protection to adjacent normal tissue structures.

## 1 Introduction

Malignancies affecting the upper abdomen, which primarily include gastric cancer, liver cancer, and pancreatic cancer, among others, present formidable clinical challenges. For many patients, surgical intervention becomes unattainable in the advanced stages of diagnosis due to various factors, including disease complexity, inoperable conditions, or the propensity for postoperative recurrence and metastasis. In the course of treatment, most patients require comprehensive radiotherapy to improve the quality of life and prognosis (1–3). Radiotherapy assumes a pivotal role in bolstering local disease control postoperatively and is an efficacious treatment modality for patients deemed unsuitable candidates for surgery. Radiotherapy is one of the most important strategies for cancer management, with approximately 50% of patients with cancer receiving radiotherapy as part of their treatment regimen, resulting in reported cure rates of up to 40% (4, 5). The evolution of radiotherapy techniques, encompassing advancements in therapeutic platforms, computational algorithms, and radiotherapy equipment, has substantially refined treatment delivery, ensuring safe and tolerable administration to target lesions while minimizing radiation exposure to surrounding critical structures (6). The role of radiotherapy in malignant-tumor treatment is currently receiving increased attention.

Postural fixation is the primary link in radiotherapy for malignant tumors and directly affects the accuracy of positioning. Inaccurate positioning leads to missed target irradiation and inadvertent exposure of critical organs to high-dose radiation, thereby precipitating adverse sequelae and treatment complications that undermine the therapeutic efficacy of radiotherapy (7). To ensure the consistency of a patient’s position from positioning to treatment, various positioning devices must be used to ensure the comfort and repeatability of the patient’s position. The precision of repeated positioning is essential to ensuring the effectiveness of radiotherapy.

The close anatomical proximity between the upper abdominal organs and the diaphragm can lead to unnecessary breathing movements, which can exacerbate setup inaccuracies. These setup errors, particularly pertinent during target delineation and treatment planning stages, often necessitate the adoption of a larger planning target volume (PTV) to ensure tumor-dose coverage, inadvertently leading to higher doses of radiation being delivered to adjacent organs at risk (8).

At present, radiotherapy for thoracic and abdominal tumors is primarily fixed utilizing a thermoplastic body film. However, the integration of both thermoplastic body film and air-bag fixation techniques (Klarity,Guangzhou,China), as used in the radiotherapy center in this study, can inhibit the range of abdominal motion during the spontaneous breathing of patients, consequently reducing the movement of upper abdominal organs. This combined approach augments the repeatability and precision of patient positioning. Use of the ABC system requires pre-treatment breathing-technique training and is contraindicated in patients with compromised pulmonary function. The BodyFIX system relies on negative pressure applied to the abdomen and thorax, which is often time-consuming for both staff and patients. In contrast, the method presented here is quick to set up, easy to use and well tolerated, leading to better patient compliance.

In this study, clinical data of 77 patients undergoing radiotherapy for upper abdominal tumors were collected. The setup errors of patients using thermoplastic body film plus air-bag fixation technology and patients using thermoplastic film fixation technology alone were analyzed using cone-beam computed tomography (CBCT).

## 2 Materials and methods

### 2.1 Patient population

This retrospective study included 77 patients with liver, pancreatic, gastric, or abdominal lymph node malignancies who underwent radiation therapy at a single institution between January 2021 and April 2023. Patients were divided into two groups based on the postural fixation technique employed. The observation group comprised 16 males and 15 females, with ages ranging from 39 to 80 years and a median age of 64 years. Among them, 25 individuals had liver cancer, 1 had pancreatic cancer, and 5 had abdominal lymph node metastasis; additionally, 11 patients underwent stereotactic body radiotherapy (SBRT), while 20 received conventional radical fractionation (CRF). Conversely, the control group consisted of 35 males and 11 females, aged between 37 and 86 years, with a median age of 65 years. Among them, 6 patients had liver cancer, 10 had pancreatic cancer, 27 were diagnosed with gastric cancer, and 3 had abdominal lymph node metastasis. All participants were treated with CRF. Main characteristics of patients and tumors are summarized in **Table 1**. This retrospective study was approved by the Ethical Committee of Huai’an First Hospital.

**Table 1:** Patient and tumor characteristics.

Variables		Value	
		n	The percent of patients(%)
Sex	Male	51	66.2%
	Female	26	33.8%
Age(years)	Median(rang)	64(37-86)	
ECOG performance status	0	15	19.5%
	1	39	50.6%
	2	23	29.9%
Number of tumors	1	72	93.5%
	2	5	6.5%
PTV volume(cm^3^)	Median(rang)	353.43(29.17-2177.72)	
Radiation therapy site	liver	31	40.3%
	gastric	27	35.1%
	pancreatic	11	14.3%
	abdominal lymph node	8	10.4%

* The values listed in the table give the number of patients (n) followed by the percent of patients (%), unless otherwise specified.

### 2.2 Patient position fixation

**Figure 1:**
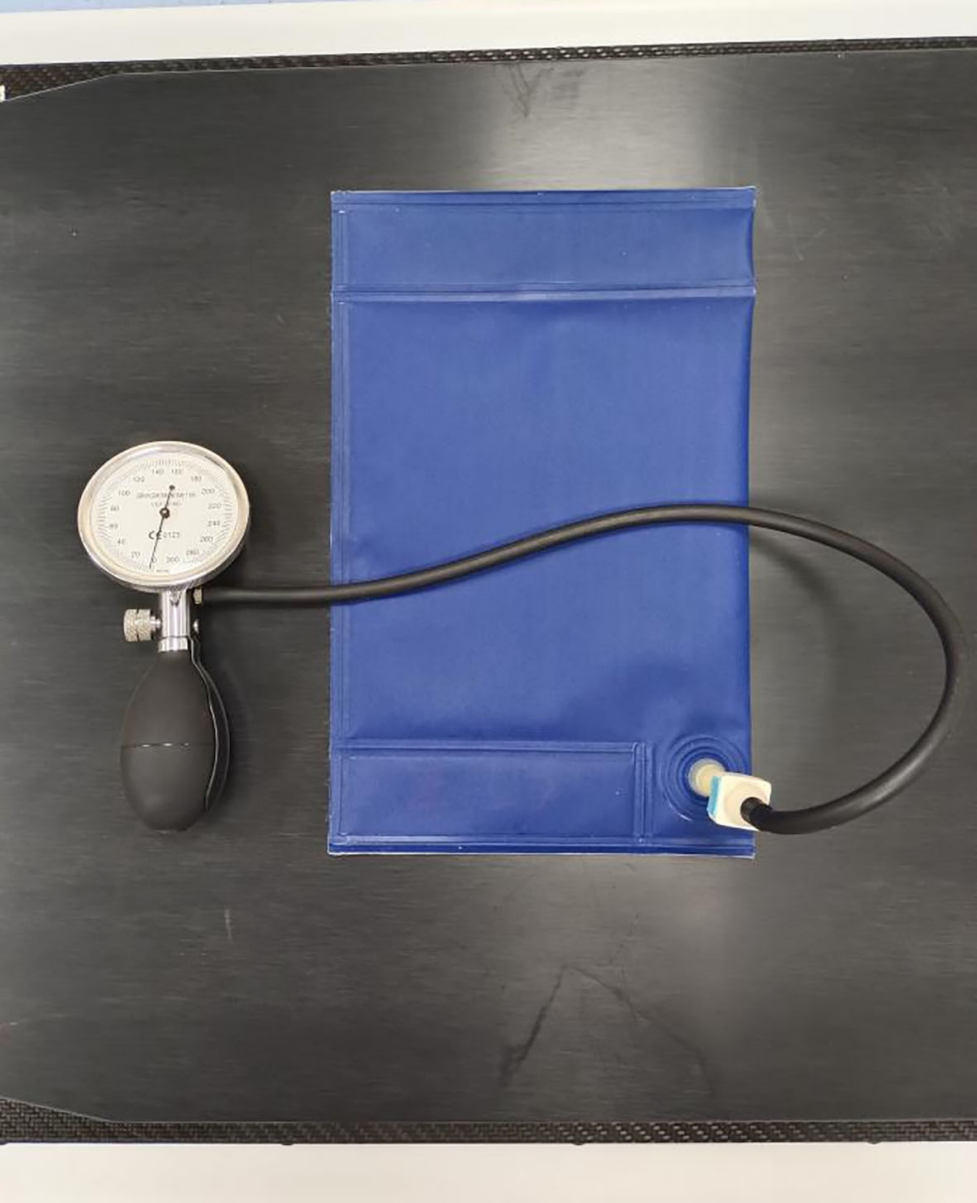
The airbag is inflated to a pressure range of 20–60 mmHg, monitored via a handheld manometer similar to the cuff of a sphygmomanometer.

Patients in the observation group assumed a supine position, with their hands crossed and elbows placed on the forehead. An uninflated air bag was attached to the skin surface under the xiphoid process. The thermoplastic body film was then contoured, with the upper boundary extending to the nipple and the lower boundary to the pelvic cavity, while ensuring the charging and discharging valves of the airbag remained exposed. After the thermoplastic body film had completely cooled, connect the air pump to the inflation valve as shown in **Figure 1**. The airbag is inflated to a pressure range of 20–60 mmHg, monitored via a handheld manometer similar to the cuff of a sphygmomanometer. The pressure in the ventilation pipe was recorded using a dial recording device, with adjustments made to ensure patient comfort and minimize abdominal breathing movements.

In contrast, participants in the control group assumed a supine posture, adopting the same arm positioning as the observation group. However, the thermoplastic body film alone was used for postural fixation. The upper boundary of the body film extended to the nipple and the lower boundary to the pelvic cavity, with the fixation finalized following thermoplastic film cooling.

### 2.3 CT scans

After the position was fixed, a CT simulator (Philips Brilliance Big Bore CT) was used for scanning. The gastric cancer scan ranged from the trachea bifurcation to the lower margin of the L4 vertebral body, while liver and pancreatic cancer scans ranged from 5 cm above the septum to the lower margin of the L4 vertebral body. Tumor-specific scans were performed with a slice thickness of 5 mm to precisely delineate lesion localization. Subsequently, CT images were transmitted to a Monaco 5.1.1 treatment planning system (Elekta, Atlanta, GA, USA). The target area was sketched by senior physicians, and the radiotherapy plan was designed by physicists. CT images containing the target area and treatment plan information were transmitted to the X-ray volume image (XVI) system integrated within the linear accelerator.

### 2.4 Setup-error measurements

Patients treated with SBRT underwent CBCT scans before each treatment, and those treated with CRF underwent CBCT scans once a week. The CBCT images were collected and subsequently transmitted to the linear accelerator XVI system to locate the CT images for registration. Grayscale registration and manual fine tuning were used to obtain the bone markers and the optimal overlap of the target tumors in the sagittal, coronal, and transverse locations of the two CT images. The setup errors of the X (left/right), Y (superior/inferior), and Z (anterior/posterior) axes were recorded. Furthermore, the displacement vector was calculated using the setup errors from all three axes ( $\text{T=}\sqrt{{\text{X}}^{2}{\text{+Y}}^{2}{\text{+Z}}^{2}}$ ).

Random and systematic errors were assessed for both the observation and control groups utilizing established methodologies as delineated in prior literature (9). Setup errors include both systematic (Σ) and random (σ) errors, where individual systematic errors denote the average of all fractional positioning discrepancies observed across a given patient group, while individual random errors represent the standard deviation of all fractional setup errors within the same patient group. Random and systematic population errors were calculated as the mean of the individual random errors and the standard deviation of the individual systematic errors, respectively (10). PTV was calculated using the formula developed by Stroom et al., expressed as MPTV = 2 Σ + 0.7 σ.

### 2.5 Statistical analysis

Statistical analyses were performed using SPSS (version 22, IBM SPSS Statistics). Data in this study are expressed as the median along with the first and third quartiles. Data analysis was performed by using the Wilcoxon rank-sum test for pairwise comparison of groups. *P* < 0.05 was considered statistically significant.

## 3 Result

### 3.1 Analysis of image registration results

**Figure 2:**
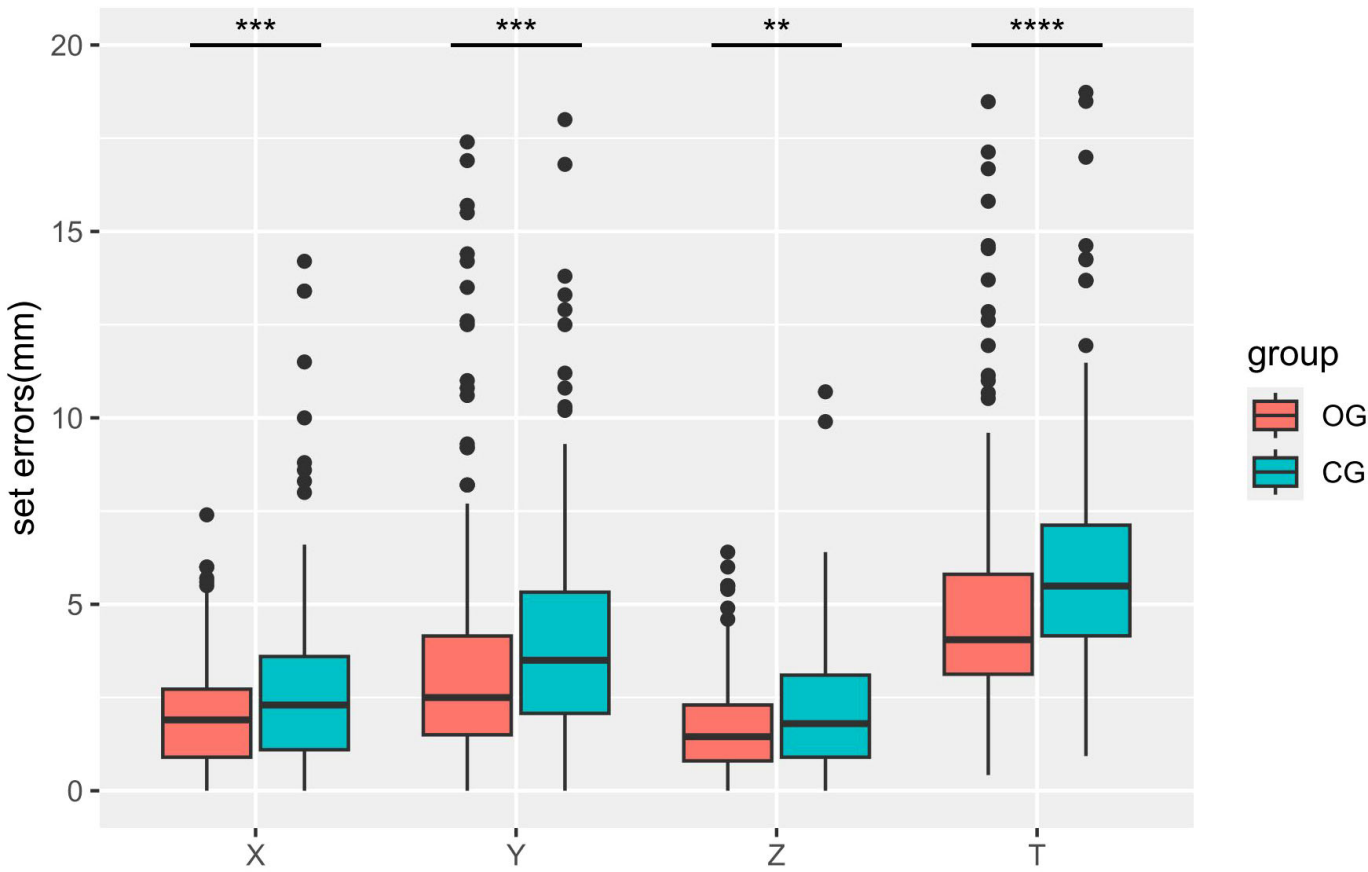
Boxplots of registration error of the observation (OG) and control (CG) groups. Box plots of registration errors in the X, Y, Z and roll (T) directions for the two groups. Boxes represent median and inter-quartile range (IQR), Where ** indicates p ≤ 0.01, *** indicates p ≤ 0.001, and **** indicates p ≤ 0.0001.

A comprehensive analysis was conducted on a total of 426 pretreatment CBCT scans, including 208 scans from the observation group and 206 from the control group. The positional errors observed were recorded in the X, Y, Z and T for each CBCT scan, as shown in **Table 2**. Additionally, **Figure 2** depicts boxplots of registration errors (in millimeters) for the two groups of patients. Notably, the three directional errors and resultant displacement vector of the observation group were smaller than those of the control group, with statistically significant differences observed (p<0.05). Furthermore, the observation group demonstrated a narrower interquartile range across all directional errors, indicative of heightened stability and reliability in positioning.

**Table 2:** Comparison of absolute error values of X, Y, Z, and T between the two groups (mm)and the median (first and third quartiles).

Characteristic	Observation group N=208	Control group N=206	P-value^1^
X	1.9(0.90,2.73)	2.3(1.1,3.6)	<0.001
Y	2.5(1.48,3.7)	3.5(2.1,5.4)	<0.001
Z	1.4(0.78,2.3)	1.8(0.9,3.1)	0.003
T	3.97(3.08,5.32)	5.51(4.18,7.25)	<0.001

^1^Wilcoxon rank-sum test; N: Image-registration times; p < 0.05, statistically significant.

### 3.2 Analysis of random errors, systematic errors, and PTV margins

**Table 3** presents the aggregated data concerning random and systematic population errors for both the observation and control groups, along with the PTV margins calculated utilizing the aforementioned formulae. Notably, the PTV margin in the superior/inferior direction exceeded those observed in the left/right direction and the anterior/posterior directions across both groups. A comparative analysis of the use of thermoplastic body film alone versus its combination with an airbag demonstrated that the combined approach significantly reduced the random overall error (σ), the system overall error (Σ), and the PTV margin.

**Table 3:** Summarized data of errors and PTV margin estimates for the observation and control groups.

Characteristic	Observation group			Control group		
	X	Y	Z	X	Y	Z
Random errors(mm)	1.93	3.1	1.59	2.85	4.13	2.26
Systematic errors(mm)	1.52	1.7	1.34	2.31	3.38	1.55
PTV margins(mm)	4.39	5.57	3.80	6.61	9.65	4.68

p < 0.05, statistically significant.

## 4 Discussion

Innovations and developments in radiation therapy techniques have necessitated more precise targeting of tumors, leading to the development of image-guided radiation therapy techniques. Enhancing the precision of radiation therapy holds promise for optimizing patient outcomes and mitigating the incidence of treatment-related toxicities. CBCT is used in linear accelerators to obtain diagnostic-quality X-ray images before treatment to assess the location of the tumor (6). In this study, CBCT was used to analyze the positioning errors for patients undergoing treatment employing thermoplastic body coupled with air-bag fixation technology, compared with those treated solely with thermoplastic body film fixation.

Position fixation in radiotherapy can effectively prevent position movement and limit the influence of respiratory movement, thereby enhancing treatment precision. The anatomical proximity of the middle-upper abdominal organs to the diaphragm can lead to unnecessary breathing movements, consequently heightening the risk of setup errors. Presently, techniques such as breath-holding maneuvers and respiratory gating methods are commonly used to manage respiratory exercise (11, 12). However, these approaches may be impractical in the case of some patients, particularly those with compromised pulmonary function. Under such circumstances, abdominal compression is usually considered (13). Abdominal compression fixation techniques offer a means to reduce tumor movement attributable to respiratory motion, notably demonstrating efficacy in patients with abdominal and pulmonary malignancies (14, 15). The technique used in this study is also an abdominal compression technique, specifically employing a combination of thermoplastic body film and an air bag. This technique can adjust the pressure exerted on the abdominal region by adjusting the inflation level of the air bag, thereby suppressing abdominal breathing and mitigating associated errors ensuring precise radiotherapy delivery.

As indicated in the data presented in **Table 2**, compared with the control group, the setup errors and displacement vector were reduced in the observation group and the difference is statistically significant. Notably, the interquartile range of the three directional setup errors and displacement vector for the combination of thermoplastic body film and air bag (X: 1.83; Y: 2.22; Z: 1.52; T: 2.24) compared with thermoplastic body film alone (X: 2.5; Y: 3.3; Z: 2.2; T: 3.07), indicates enhanced stability and reliability with the combined approach. As reported by Catherine et al. (16), the setup errors for BodyFIX without wrap were X:1.87 (1.25); Y: 2.55 (1.8); Z:2.05 (2.34), whereas the present study documents setup errors for the combination of thermoplastic body film and airbag as X:1.9 (1.83); Y: 2.5 (2.22); Z:1.4 (1.52). Notably, directional errors along the X (left/right) and the Y (superior/inferior) axes are similar between the two methods, while substantial advantages are observed in the direction of the Z (anterior/posterior) axis with the combination technique employed herein. This discrepancy may be because the method used in this study can accurately control the pressure of the air bag, generating significant intra-abdominal pressure. Conversely, the BodyFIX system operates on negative pressure within the entire chest and abdominal cavity, which is slightly less than the pressure exerted on the abdomen in this study. This may be the reason why the combined use of thermoplastic film and an air bag reduces the forward and backward displacement errors of the abdominal cavity. Furthermore, in clinical practice, the prolonged positioning time and limited patient compatibility associated with the BodyFIX system pose challenges, particularly for patients unable to sustain extended periods of cooperation. Before using the ABC system, patients must undergo respiratory technique training. They are required to actively inhale to a specific threshold and maintain that breath for a defined duration, during which irradiation is performed. Multiple repetitions are necessary to complete the full irradiation process, which is time-consuming and particularly challenging for patients with compromised lung function.However, this fixation procedure is also lengthy. In contrast, the method proposed in this study demonstrates strong clinical applicability and can be more readily implemented in facilities lacking the ABC or BodyFix systems. It utilizes only standard thermoplastic body film and inflatable airbags, thereby reducing equipment costs and enhancing patient comfort, which in turn improves treatment compliance.

Analysis of **Table 2** and **Figure 2** reveals a notable predilection for errors to manifest predominantly in the superior/inferior direction, which is consistent with previously reported results (Zhong et al. and Yang et al.) (17, 18). This finding may be due to the greater respiratory movement in the superior/inferior direction. Existing studies report liver displacement of 1 to 2 cm in the superior/inferior plane during breathing, with comparatively lesser displacement observed along the anterior/posterior and left/right direction (19). The superior/inferior direction setup error exhibited a more pronounced reduction with the employment of thermoplastic body film combined with an airbag, indicative of the efficacy of this combined approach in attenuating the influence of respiratory motion. The reduction in setup errors has great significance for precision radiotherapy. While enhancing the precision of radiotherapy, it concurrently enables the administration of higher single radiation doses, thereby fostering improved local control rates for tumors.

The results depicted in **Table 3** indicate that the combination of thermoplastic body film and an air bag reduced the random and systematic population errors, with the random errors generally surpassing systematic errors, in line with the results reported by Dawson et al. (20) Considering the effects of setup errors, standard treatment techniques require large margins in the gross tumor volume to ensure adequate doses are given to the tumor throughout the course of treatment. Notably, the combined use of thermoplastic film and an air bag greatly reduced PTV margins, a reduction of about 42%, especially in the superior/inferior direction. During the delineation of the target volume, clinicians take into account factors such as tumor mobility, respiratory dynamics, and setup inaccuracies. Consequently, PTV margins are delineated beyond the clinical target volume to encompass potential uncertainties, ensuring maximal tumor coverage and requisite radiation dosage delivery. The expansion of the irradiation range can have side effects and increase the dose to normal tissues. Reducing the PTV margin can decrease the dose delivered to normal organs and tissues while maintaining the prescribed dose to the target. This reduction helps to limit the irradiation area, which is crucial for protecting normal tissues, minimizing radiation-induced toxicity, and preserving the function of regenerative organs such as the liver. Furthermore, reducing the PTV margin not only allows dose escalation to the tumor, thereby increasing its biological response, but also decreases the dose to surrounding normal tissues. This reduction can lower the risk of radiation-induced toxicities and better preserve adjacent organ function.

The limitation of the current retrospective study stems from the constrained sample size, which precluded a comprehensive analysis of setup errors specific to upper abdominal tumors, namely liver cancer, pancreatic cancer, gastric cancer, and lymph node radiotherapy. Group heterogeneity may influence the study outcomes. Therefore, a subsequent step involves expanding the number of registered cases to enable a stratified analysis of positioning errors based on tumor location and treatment modality. Secondly, CBCT scans were conducted on a daily basis in the SBRT group, whereas they were performed only once weekly in the CRF group, potentially introducing bias into the results. This limitation is further compounded by the retrospective nature of the analysis. Future research should prioritize prospective studies and adopt more standardized imaging protocols to further minimize the impact of this bias. Thirdly, the observation group comprised 11 SBRT and 20 CRF patients, whereas the control group contained 46 CRF patients only. After stratification the SBRT subgroup became too small for meaningful statistics. A prospective study with balanced subgroup sizes is planned to overcome this drawback and to verify whether the observed improvements are indeed attributable to the immobilisation technique. Additionally, the absence of intra-fraction motion assessment represents another limitation of this study. Future studies should focus on whether the combination of thermoplastic body film and an air bag for postural fixation during treatment can provide better stability for radiation therapy.

## 5 Conclusion

The results of this study indicated that the combination of thermoplastic body film and an air bag for postural fixation during mid-upper abdominal radiotherapy provided superior positioning accuracy and repeatability compared to the utilization of thermoplastic body film alone. This approach is straightforward to implement, easily adoptable in clinical settings, and has the potential to significantly enhance the practicality and applicability of radiotherapy for this anatomical region. Future prospective studies with larger and more homogeneous cohorts are warranted to validate these findings.

## Data Availability

The original contributions presented in the study are included in the article/supplementary material. Further inquiries can be directed to the corresponding author.
